# Anaemia and its determinants among young children aged 6–23 months in Ethiopia (2005–2016)

**DOI:** 10.1111/mcn.13082

**Published:** 2020-09-23

**Authors:** Helen Heinrichs, Bilal Shikur Endris, Tariku Dejene, Geert‐Jan Dinant, Mark Spigt

**Affiliations:** ^1^ Faculty of Health, Medicine and Life Sciences Maastricht University Maastricht The Netherlands; ^2^ School of Public Health Addis Ababa University Addis Ababa Ethiopia; ^3^ Center for Population Studies Addis Ababa University Addis Ababa Ethiopia; ^4^ School CAPHRI, Care and Public Health Research Institute Maastricht University Maastricht The Netherlands; ^5^ General Practice Research Unit, Department of Community Medicine The Arctic University of Tromsø Tromsø Norway

**Keywords:** anaemia, children aged 6–23 months, determinants, DHS, Ethiopia, prevalence, public health

## Abstract

Anaemia in children remains a significant public health threat. Recent numbers from Ethiopia showed that more than two‐thirds of children under the age of 2 years were anaemic. This study aimed to investigate the determinants of anaemia throughout Ethiopia over 11 years, making use of the Ethiopian Demographic and Health Survey (EDHS) rounds 2005, 2011 and 2016. The EDHS made it possible to use data on blood tests and detailed questionnaires among infants and young children. Multivariable logistic regression was applied to assess the association of anaemia and different immediate and underlying determinants. A total of 7,324 children aged 6–23 months were included in the analysis, with prevalences of anaemia being 71% in 2005, 61% in 2011 and 72% in 2016. The following determinants were significantly associated with childhood anaemia throughout the entire period: children younger than 1 year, anaemic mothers and those growing up in pastoralist regions. Risk factors such as diet and infections were consistently not significantly associated with anaemia. Given the tremendous adverse health effects of anaemia in young children, urgent action is needed. Hence, this study recommends nationwide multisectoral interventions targeting pastoralist regions, maternal and child health, screening and treatment of risk groups that could reduce the prevalence of anaemia.

**Key messages**

- Among children aged 6–23 months in Ethiopia, anaemia remained a severe public health concern, and no progress was made from 2005 to 2016.
- The key determinants of anaemia among young children aged 6–23 months are younger age (6–11 months), anaemic mothers and growing up in pastoralist regions.
- Given the lack of anaemia reduction in Ethiopia, investing in large‐scale evidence‐based and multisectoral programmes is required after evaluating national anaemia prevention gaps.

## 1 INTRODUCTION

Anaemia remains a significant global health concern with over one‐quarter of the world's population affected by the condition (Balarajan, Ramakrishnan, Ozaltin, Shankar, & Subramanian, 2011). Developing countries, in particular, Africa and South‐East Asia, account for over 89% of the global anaemia burden (Kassebaum, 2016; WHO, 2015). Aside from disproportionately affecting these regions, anaemia prevalence differs by age group. In particular, young children under the age of 5 years account for over 47% of the total burden (Balarajan et al., 2011), with one of the highest prevalence existing in Ethiopia (World Health Organization [WHO], 2015). The Ethiopian Demographic and Health Survey (EDHS) 2016 reported that 57% of the children under the age of 5 years were anaemic. Levels were highest in those aged 6–23 months, with 72% of these children having anaemia (Central Statistical Agency/Ethiopia [CSA] and ICF, 2017).

Anaemia results in a clinical syndrome, primarily manifesting in fatigue (Lanzkowsky, 2016). In children, the long‐term consequences are of significant concern. These are multifaceted and, if left untreated, can result in impaired growth, poor cognitive and motor development, which results in children being less attentive and exploratory (Lozoff et al., 2006; Ngure et al., 2014). Additionally, anaemia leaves children with increased susceptibility to infections (Abebe et al., 2018; Muchie, 2016). As such, diagnosis and treatment of anaemia in children should be of primary concern. Given the distinct period of rapid growth experienced by children aged 6–23 months, this age group is particularly at risk and therefore, assuring adequate iron intake is of utmost importance (Malako, Teshome, & Belachew, 2018).

The condition is characterized by a decreased amount of red blood cells or low haemoglobin levels in the blood (Desalegn, Mossie, & Gedefaw, 2014). Various studies have documented several factors associated with anaemia (Adish, Esrey, Gyorkos, & Johns, 1999; Desalegn et al., 2014; Ejigu, Wencheko, & Berhane, 2018; Reithinger et al., 2013). These showed that anaemia is multifactorial, however primarily of nutritional nature, with low intake and absorption of iron from the diet as its dominant cause (Desalegn et al., 2014). In Ethiopia, anaemia sometimes manifests secondary to another disease, including infectious diseases and intestinal parasites such as malaria and hookworm. The malaria virus, which infects red blood cells, results in the rupture and loss of these cells, eventually leading to anaemia (Barreiro et al., 2017; Desalegn et al., 2014; Girum, Shumbej, & Shewangizaw, 2019; Reithinger et al., 2013; Taffese et al., 2018; White, 2018). Children of breastfeeding age are at a particularly high risk of developing anaemia. Studies have suggested that the stored gestational iron in the mother's breast milk may be insufficient (Li, Liang, Liang, Shi, & Han, 2019). As such, in these children, the consumption of foods high in iron, for example, red meat, vegetables and fruits is essential for the prevention of iron deficiency, whereas consuming tea and foods high in fibre should be avoided (De la Cruz‐Góngora, Villalpando, Rebollar, Shamah‐Levy, & Humarán, 2012; Li et al., 2019; United States Agency for International Development [USAID], 2013). In Ethiopia, adequate nutrition is of real concern. Data from the 2016 DHS showed that only 7% of all children aged 6–23 months reached the minimum standard of the infant and young child feeding (IYCF) minimum acceptable diet (MAD). This recommended standard of diet includes breastfeeding and diversity in nutrient‐rich foods; a lack of which may explain the high prevalence of anaemia in this context (CSA and ICF, 2017). Socio‐demographic and economic factors may also contribute to the prevalence of anaemia, with higher levels in those with poorer education and socioeconomic status. Furthermore, studies indicate that the proportion of anaemia varies across geographical regions, with children living in rural areas being more at risk than those living in urban areas (Abebe et al., 2018; Ejigu et al., 2018). These risk factors are well documented; however, identifying the contribution of risk factors is needed to determine the most critical predictors of anaemia in Ethiopia, which then can be targets of interventions.

To develop highly effective interventions and policies targeting a reduction in the prevalence of anaemia, it is essential to summarize key determinants, temporal trends and geographic patterns within Ethiopia. As such, anaemia has been prioritized at the global level through the second World Health Organization (WHO) Global Nutrition Target for 2025 (WHO, 2014) and nationally through the Ethiopian Health Extension Program (Federal Ministry of Health Ethiopia, n.d.; Workie, Ramana,, & The World Bank, 2013). Despite this, efforts focused on tackling anaemia in children under 2 years are currently insufficient.

In spite of its magnitude in Ethiopia, limited evidence is available regarding anaemia's broad set of determinants. As previous research was mostly focusing on single time points, there is a scarcity of information on the patterns of determinants of anaemia over time. Hence, we present a comprehensive overview of anaemia in entire Ethiopia, validating its multifactorial set of determinants and covering the period of 2005 to 2016, the period over which haemoglobin data were available. Therefore, this study aims to examine the trends and drivers of anaemia by identifying geographical patterns and associated determinants among children aged 6–23 months in Ethiopia for over 10 years.

## 2 METHODS

### 2.1 Data sources and collection

National‐based data on the prevalence of anaemia in children were used from the EDHS rounds 2005, 2011 and 2016. The surveys covered all administrative areas in Ethiopia, using a stratified, two‐stage cluster sampling design (CSA & ICF, 2012, 2017; CSA & ORC Macro, 2006). Across the three surveys, a total of 50,470 households were selected, from which 93% were successfully interviewed. This resulted in a total of 22,568 children being tested for anaemia. This study was restricted to children between 6 and 23 months. Consequently, we used the data of 1,290 children in 2005, 2,970 in 2011 and 3,064 in the year 2016.

Further information on the methodology can be found elsewhere (CSA and ICF, 2012, 2017; CSA and ORC Macro, 2006).

### 2.2 Anaemia and its determinants

Appendix SA1 displays the variables included at baseline, and Appendix SB1 visually presents the relation of childhood anaemia determinants in the form of a conceptual framework. The determinants have been chosen based on literature search and available data in the EDHSs and were organized in line with the established United States Agency for International Development (USAID) conceptual framework for anaemia (CSA and ICF, 2012, 2017; CSAand ORC Macro, 2006; USAID, 2013). The variables were coded as follows:

#### 2.2.1 Outcome variable

Blood samples were drawn from a drop of blood taken from the palm side of the end of a finger, and in the case of infants younger than 12 months, blood was taken from the heel prick. Anaemia status was defined as mild, moderate and severe anaemia. For this study's purpose, this was recoded into a dichotomous variable, having anaemia or not. Any blood haemoglobin count below 11 g/dl was considered anaemic.

#### 2.2.2 Immediate determinants

Participants were asked whether their child was infected with any of the three most common childhood illnesses 2 weeks preceding the survey: fever, diarrhoea and signs of acute respiratory infection. Nutritional deficiencies were identified by asking mothers about the food the children ate the day preceding the survey: flesh foods, including meat, fish, poultry and liver/organ meats; Vitamin A‐rich fruits and vegetables; legumes; milk and dairy. A minimum dietary diversity variable was created to identify whether the child ate at least four out of seven food groups as recommended and defined by the WHO (WHO, 2009). Lastly, mothers were asked whether they were breastfeeding or not.

#### 2.2.3 Underlying determinants

The two variables of latrine facility and drinking water sources were classified into improved or unimproved based on the definitions by the WHO/United Nations Children's Fund (UNICEF) Joint Monitoring Program (JMP) for Water Supply and Sanitation (WHO, 2017). Slight changes in classifications across the different surveys were taken into account. We made a distinction between one or two births and more than two births, as increased intervals between pregnancies are associated with an increase in haemoglobin level and a decrease in adverse health outcomes (Afeworki, Smits, Tolboom, & van der Ven, 2015; Conde‐Agudelo, Rosas‐Bermudez, Castano, & Norton, 2012). Additionally, the women were asked whether they took the recommended minimum of 90 iron tablets or syrup during the pregnancy of their last born child and whether they received a minimum of four antenatal care visits according to the WHO (Croft, Marshall, & Allen, 2018). Other determinants were identified with the questions whether the child received vitamin A supplements or deworming in the 6 months preceding the interview. Lastly, we checked whether the national routine immunization had been completed in all children at the intended time. The routine immunization schedule in Ethiopia comprises six vaccine‐preventable diseases, measles, diphtheria, pertussis, tetanus and tuberculosis (Federal Ministry of Health, 2015).

#### 2.2.4 Basic causes

The nutritional status among children was assessed by applying three indices: height‐for‐age, weight‐for‐height and weight‐for‐age, each providing different information on growth and body composition (CSA & ICF, 2017). To summarize essential attributes of the children and mothers, we included some baseline characteristics: gender and age of the children, anaemia status and age of the mothers and the educational and geographical background of the mothers and their partners. The regions were distributed as follows: Afar, Somali, Gambela and Benishangul‐Gumuz as pastoralist regions (also referred to as pastoralist and emerging regions in Ethiopian context), the regions of Tigray, Amhara, Oromiya and the Southern Nations, Nationalities and People's Region (SNNPR) represent the agrarian region, and the three cities Harari, Dire Dawa and Addis Ababa were combined (Federal Ministry of Health Ethiopia, n.d.). Also, the wealth index was added as an appropriate measure of a household's cumulative living standard. This index consists of data on several selected household assets such as water access and sanitation facilities, televisions and bicycles. The DHS distributes the population into five wealth quintiles ranging from the poorest to the wealthiest (CSA & ICF, 2017).

### 2.3 Data analysis

The statistical analysis of the data was performed using STATA 15. To ensure a representative sample, we applied complex sample design weightings to all analyses (Croft et al., 2018). Descriptive statistics were used to analyse baseline characteristics to provide an overall picture of the sample. Pearson's correlation was run to determine the relationship between all predictor variables. Positive correlations were found, but were adjusted for in the following analyses by using the robust estimator (Do Cameron & Miller, 2015). An exploratory cross‐tabulation of anaemia prevalence and its determinants was performed to guide further analysis. We used multivariable logistic regression models to investigate the relationship between anaemia and the selected predictors per year. Independent variables were deleted by using the ‘backward’ elimination principle. For all independent variables, the nonrisk factor was considered the reference category. A significance level of 0.05 was chosen for all analyses.

The socioeconomic gradient was investigated using the wealth index and corresponding wealth quintiles. In this study, the Erreygers concentration index (CI) using wealth as a rank variable was computed for each year (Ambel et al., 2017). The CI is a value bounding between −1 and +1. A negative index indicates that poorer households disproportionately bear the burden of anaemia, whereas a positive value indicates that wealthier households are more affected (Bilger, Kruger, & Finkelstein, 2017; Cai, Coyte, & Zhao, 2017).

### 2.4 Ethical Considerations

The study received ethical approval by the Health, Ethics & Society of the Faculty of Health, Medicine and Life Sciences at Maastricht University. Ethical clearances for the surveys were provided by the EHNRI Review Board, the National Research Ethics Review Committee, the ORC Macro Institutional Review Board in Calverton, USA, the Institutional Review Board of ICF International and the United States Centers for Disease Control and Prevention.

## 3 RESULTS

### 3.1 Baseline characteristics

Table 1 summarizes the baseline characteristics of the three samples included in the study. Haemoglobin measures were available for 7,324 children aged 6–23 months. The samples revealed that anaemia was a persistent problem affecting a large proportion, with worrisome values of 71%, 61% and 72% over the different years.

**TABLE 1:** Baseline characteristics of the children and their caretakers per year (*N* = 7,324)

		EDHS 2005 (*N* = 1,290)	EDHS 2011 (*N* = 2,970)	EDHS 2016 (*N* = 3,064)
Variables		%^*^ (*N*)	%^*^ (*N*)	%^*^ (*N*)
Anaemia (child)	No	28.8 (371)	39.1 (1,161)	28.1 (861)
	Yes	71.2 (918)	60.9 (1,809)	71.9 (2,204)
Sex (child)	Male	48.9 (631)	51.3 (1,522)	47.5 (1,455)
	Female	51.1 (659)	48.7 (1,447)	52.5 (1,610)
Age (child/months)	6–11	32.9 (425)	36.1 (1,072)	34.0 (1,043)
	12–23	67.1 (865)	63.9 (1,898)	66.0 (2,022)
Nutrition status (child)				
Stunting	No	58.9 (760)	64.6 (1,919)	68.1 (2,084)
	Yes	41.1 (530)	35.4 (1,051)	31.9 (975)
Wasting	No	85.5 (1,103)	86.0 (2,553)	87.1 (2,668)
	Yes	14.5 (187)	14.0 (417)	12.9 (394)
Underweight	No	62.0 (800)	74.3 (2,206)	79.1 (2,421)
	Yes	38.0 (490)	25.7 (764)	20.9 (641)
Age (women/years)	15–19	6.2 (78)	6.5 (186)	5.3 (156)
	20–34	68.3 (856)	74.4 (2,143)	74.9 (2,202)
	35–49	25.5 (320)	19.2 (553)	19.8 (582)
Anaemia (women)	No	72.2 (894)	83.4 (2,387)	69.7 (2,027)
	Yes	27.8 (345)	16.6 (475)	30.4 (883)
Education (women)	No	77.4 (970)	68.4 (1,971)	61.2 (1,801)
	Primary	18.4 (231)	27.6 (797)	31.4 (922)
	Secondary/higher	4.2 (53)	4.0 (114)	7.4 (218)
Education (partner)	No	56.4 (698)	49.2 (1,400)	44.1 (1,223)
	Primary	33.3 (412)	42.6 (1,213)	42.6 (1,181)
	Secondary/higher	10.3 (127)	8.2 (232)	13.3 (367)
Marital status	Married/living together	94.5 (1,185)	93.3 (2,689)	95.0 (2,794)
	Widowed/divorced	3.9 (49)	4.7 (135)	3.1 (90)
	Single	1.6 (20)	2.0 (58)	1.9 (56)
Regions	Tigray	6.6 (85)	6.7 (200)	7.6 (233)
	Afar	0.8 (10)	0.8 (24)	1.0 (30)
	Amhara	22.0 (283)	21.9 (650)	19.3 (592)
	Oromiya	39.7 (512)	43.6 (1,296)	43.8 (1,342)
	Somali	2.6 (34)	2.3 (69)	3.7 (113)
	Ben‐gum.	1.0 (13)	1.2 (35)	1.0 (30)
	SNNPR	25.4 (328)	20.8 (619)	20.8 (636)
	Gambela	0.2 (3)	0.3 (9)	0.2 (7)
	Harari	0.2 (3)	0.2 (6)	0.2 (6)
	Addis Ababa	1.2 (16)	1.8 (54)	2.1 (65)
	Dire Dawa	0.3 (4)	0.4 (9)	0.4 (11)
Rural vs. urban	Pastoralist	4.6 (60)	4.6 (137)	5.9 (180)
	Agrarian	93.7 (1,208)	93.1 (2,764)	91.4 (2,802)
	Cities	1.7 (22)	2.3 (69)	2.7 (82)
Wealth	Poorest	22.3 (288)	24.1 (716)	22.7 (694)
	Poor	21.1 (272)	22.6 (672)	21.2 (648)
	Middle	22.3 (287)	20.7 (614)	22.8 (699)
	Wealthy	19.1 (247)	18.4 (546)	19.2 (588)
	Wealthiest	15.2 (196)	14.2 (422)	14.2 (436)

*Note*: Stunted = (severely) stunted; wasted = (severely) wasted; Ben‐Gum.: Benishangul‐Gumuz.

* *p* < 0.05.

### 3.2 Prevalence of anaemia by determinants

Table 2 shows the prevalence of anaemia by each determinant. The higher prevalence of anaemia was observed in children younger than 1 year, those who grew up in pastoralist regions such as Somali or Afar, those whose mothers were anaemic, malnourished children and those whose parents attained lower education. Though not always proven statistically significant, the prevalence of anaemia was higher in both 2005 and 2016 for almost all determinants compared with the year 2011.

**TABLE 2:** Percentage of children aged 6–3;23 months classified as having anaemia by independent variable per year

		EDHS 2005		EDHS 2011		EDHS 2016	
		Anaemia prevalence		Anaemia prevalence		Anaemia prevalence	
Variables		Anaemic (%)	95% CI	Anaemic (%)	95% CI	Anaemic (%)	95% CI
Dependent variable							
Anaemia		71.2	67.8; 74.4	60.9	57.9; 63.8	71.9	68.7; 74.9
Underlying determinants: socio‐demographic and socioeconomic							
Sex (child)	Male	75.0^*^	70.2; 79.2	60.7	56.5; 64.7	73.2	69.0; 77.0
	Female	67.6^*^	62.9; 72.0	61.1	57.1; 65.0	70.7	66.6; 74.5
Age (child)	6–11	75.4	69.5; 80.5	66.6^*^	61.9; 71.0	77.2^*^	72.5; 81.2
	12–23	69.1	65.0; 73.0	57.7^*^	54.0; 61.3	69.2^*^	65.6; 72.6
Stunting	No	68.4^*^	63.8; 72.7	59.9	56.4; 63.3	71.2	67.5; 74.7
	Yes	75.2^*^	70.0; 79.8	62.7	57.9; 67.3	73.3	68.8; 77.4
Wasting	No	69.9	66.2; 73.4	60.3	57.1; 63.4	70.3^*^	66.9; 73.4
	Yes	78.7	69.5; 85.8	64.6	57.4; 71.2	82.8^*^	76.4; 87.8
Underweight	No	66.6^*^	62.1; 70.9	59.6	56.2; 62.9	70.0^*^	66.3; 73.5
	Yes	78.7^*^	73.4; 83.1	64.8	59.2; 70.0	79.0^*^	73.5; 83.5
Age (w.)	15–19	84.2	71.4; 92.0	64.3	53.9; 73.6	79.0	68.0; 86.8
	20–34	71.5	67.4; 75.3	61.1	57.5; 64.5	72.3	68.6; 75.7
	35–49	69.3	62.0; 75.7	59.3	53.8; 64.6	68.6	62.9; 73.8
Anaemia (w.)	No	69.1^*^	65.0; 73.0	59.6^*^	56.4; 62.7	68.2^*^	64.7; 71.6
	Yes	79.1^*^	72.8; 84.3	67.1^*^	60.5; 73.0	80.2^*^	75.5; 84.2
Education (w.)	No	73.4	69.6; 76.9	63.1^*^	59.7; 66.5	72.6^*^	68.7; 76.2
	Primary	68.9	60.1; 76.5	57.8^*^	52.4; 63.0	74.1^*^	69.0; 78.7
	Secondary/higher	53.9	36.6; 70.3	45.7^*^	34.5; 57.2	57.3^*^	48.8; 65.4
Education (p.)	No	74.3	69.7; 78.4	61.6^*^	57.3; 65.7	72.5	67.9; 76.6
	Primary	68.2	61.8; 74.1	63.1^*^	58.4; 67.6	73.3	68.1; 77.8
	Secondary/higher	67.5	56.3; 77.0	48.3^*^	38.2; 58.6	63.9	57.2; 70.2
Marital status	Married/living t	71.1	67.5; 74.3	60.4	57.3; 63.5	71.8	68.6; 74.8
	Wid./div.	82.2	63.8; 92.4	71.0	59.9; 80.0	72.0	54.6; 84.6
	Single	87.3	55.7; 97.4	62.1	43.4; 77.6	78.1	57.7; 90.3
Region	Tigray	77.3^*^	68.8; 84.0	60.2^*^	52.9; 67.0	73.6^*^	67.4; 78.9
	Afar	77.7^*^	63.1; 87.7	88.0^*^	83.5; 91.5	88.7^*^	83.9; 92.3
	Amhara	72.6^*^	64.8; 79.2	55.4^*^	48.4; 62.1	65.5^*^	59.3; 71.3
	Oromiya	76.1^*^	70.1; 81.1	66.8^*^	61.6; 71.7	77.6^*^	72.0; 82.3
	Somali	94.0^*^	83.2; 98.0	76.3^*^	66.7; 83.9	91.1^*^	87.2; 93.9
	Ben‐Gum.	58.8^*^	49.8; 67.3	60.9^*^	53.2; 68.1	59.8^*^	52.5; 66.6
	SNNPR	59.1^*^	52.8; 65.1	52.5^*^	47.1; 57.9	62.4^*^	55.4; 68.9
	Gambela	70.7^*^	50.1; 85.3	67.3^*^	55.5; 77.2	69.3^*^	60.7; 76.8
	Harari	79.3^*^	67.8; 87.5	73.4^*^	66.3; 79.5	81.4^*^	73.8; 87.2
	Addis Ababa	61.2^*^	40.8; 78.3	47.2^*^	37.3; 57.2	61.9^*^	53.3; 69.8
	Dire Dawa	75.4^*^	58.5; 87.0	78.6^*^	70.3; 85.1	84.5^*^	75.2; 90.7
Rural vs. urban	Pastoralist	82.7^*^	76.0; 87.8	73.8^*^	68.3; 78.7	84.6^*^	81.2; 87.5
	Agrarian	70.7^*^	67.1; 74.1	60.5^*^	57.2; 63.6	71.3^*^	67.7; 74.5
	Cities	65.7^*^	50.6; 78.2	53.6^*^	46.0; 61.0	66.4^*^	59.3; 72.8
Wealth	Poorest	78.5^*^	72.1; 83.7	61.3^*^	55.8; 66.6	81.5^*^	76.1; 85.9
	Poor	76.2^*^	68.4; 82.6	65.9^*^	60.5; 70.7	72.2^*^	66.9; 77.0
	Middle	73.5^*^	65.9; 79.9	62.9^*^	56.5; 69.0	70.0^*^	62.9; 76.3
	Wealthy	59.0^*^	50.9; 66.6	60.3^*^	53.5; 66.7	68.3^*^	61.6; 74.3
	Wealthiest	65.7^*^	56.8; 73.7	50.0^*^	41.7; 58.3	64.1^*^	57.6; 70.1
Immediate determinants: nutritional deficiencies							
Duration breastfeeding	Ever, not currently	60.5	45.7; 73.6	61.3	50.6; 71.0	75.1	67.7; 81.2
	Never breastfed	65.5	16.3; 94.9	82.5	54.4; 94.9	69.1	50.1; 83.3
	Still breastfeeding	72.7	69.1; 75.9	60.8	57.5; 63.9	71.5	68.2; 74.7
Flesh food	No	71.8	68.3; 75.1	61.1	58.0; 64.1	72.5	69.1; 75.6
	Yes	72.6	58.9; 83.1	59.2	49.3; 68.5	65.8	56.8; 73.9
Legumes	No	72.9	68.8; 76.6	61.9	58.7; 65.0	73.9^*^	70.4; 77.2
	Yes	68.8	61.7; 75.1	56.8	49.9; 63.4	64.6^*^	58.4; 70.3
Vegetables and fruits	No	73.7^*^	70.0; 77.1	62.4^*^	59.3; 65.5	73.3	69.6; 76.6
	Yes	62.7^*^	53.9; 70.8	50.2^*^	43.1; 57.3	67.9	61.7; 73.5
Milk	No	71.2	66.6; 75.4	61.6	58.1; 64.9	71.9	68.5; 75.2
	Yes	72.8	67.1; 77.9	58.9	53.0; 64.5	72.2	66.2; 77.5
Dairy	No	71.3	67.6; 74.7	60.3	57.0; 63.5	71.1	67.8; 74.2
	Yes	75.6	65.1; 83.7	67.6	57.4; 76.4	75.4	68.5; 81.1
Dietary diversity	Below 4	71.2	67.7; 74.4	61.1	58.2; 64.0	72.4	69.1; 75.4
	4 and above	75.8	41.3; 93.3	48.9	29.2; 68.9	66.3	55.6; 75.6
Iron‐rich food	No	72.8	68.2; 76.9	63.6^*^	60.1; 66.9	75.3^*^	71.3; 78.9
	Yes	68.7	63.2; 73.7	55.0^*^	50.0; 60.0	66.9^*^	62.3; 71.2
Iron‐inhibiting food	No	71.5	66.9; 75.7	60.7	56.9; 64.3	70.9	67.3; 74.1
	Yes	72.1	66.6; 77.0	61.7	56.3; 66.8	74.3	68.9; 79.1
Immediate determinants: infections							
Fever	No	72.1	68.1; 75.8	61.2	57.9; 64.5	70.6^*^	66.9; 74.0
	Yes	70.8	64.0; 76.8	60.2	54.5; 65.7	77.5^*^	71.8; 82.3
Cough	No	72.9	69.1; 76.3	62.1	58.6; 65.4	71.6	68.0; 75.0
	Yes	67.8	60.7; 74.3	57.6	51.8; 63.2	72.9	67.4; 77.7
Diarrhoea	No	69.3^*^	65.0; 73.4	61.5	58.2; 64.6	71.6	67.9; 75.1
	Yes	77.3^*^	71.5; 82.2	59.7	54.1; 65.0	73.0	67.4; 78.0
Underlying determinants: Inadequate prevention/treatment and maternal care							
Deworming	No	72.3	68.7; 75.6	61.3	58.1; 64.3	71.2	67.9; 74.3
	Yes	64.3	40.6; 82.6	58.7	50.0; 66.8	76.3	67.1; 83.6
Full immunization	No	70.4	66.5; 74.0	60.8	57.5; 64.1	71.1	67.3; 74.5
	Yes	74.5	66.0; 81.6	61.2	55.2; 66.9	74.4	69.2; 79.0
Vitamin A supplementation	No	71.8	67.1; 76.1	63.1	59.2; 66.9	72.3	68.7; 75.6
	Yes	72.1	66.8; 76.8	59.3	55.5; 62.9	71.6	67.0; 75.4
ANC visits	<4	72.4	68.7; 75.9	61.1	57.7; 64.5	72.6	68.6; 76.2
	≥4	68.9	59.6; 76.9	56.9	50.4; 63.3	70.0	65.6; 74.0
Nr. of births	1 or 2	71.1	67.4; 74.5	60.4	57.3; 63.5	71.2	68.2; 74.1
	More than 2	72.3	60.8; 81.4	65.5	57.7; 72.5	79.4	68.1; 87.5
Underlying determinants: water, hygiene and sanitation							
Drinking water	Improved	71.5	66.7; 75.9	58.2	53.8; 62.5	70.7	67.3; 73.9
	Unimproved	71.0	65.8; 75.7	63.2	59.3; 66.9	73.6	68.6; 78.0
Toilet facility	Improved	67.0	56.6; 76.0	54.1	45.8; 62.2	71.8	65.3; 77.5
	Unimproved	71.5	67.9; 75.0	61.8	58.7; 64.9	71.9	68.5; 75.1

*Note*: Variables are defined as in the methods section.

* *p* < 0.05.

### 3.3 Factors associated with anaemia

Table 3 shows the results of the multivariable logistic regression analysis of children's anaemia status in 2005, 2011 and 2016. The analysis showed that the odds of anaemia was significantly higher for 6–11 months old across all three EDHSs (odds ratio [OR] = 1.7 [1.0–2.8]; OR = 1.7 [1.2–2.2]; OR = 1.5 [1.1–2.1]). Children whose mothers were anaemic were more likely to suffer from anaemia in 2005 and 2016 (OR = 2.1 [1.4–3.2]; OR = 1.7 [1.2–2.3]) and those living in pastoralist regions in 2011 and 2016 (OR = 1.8 [1.4–2.4]; OR = 1.5; [1.1–2.1]). Household wealth was also found to be associated with a higher prevalence of anaemia, with children from households in the wealthy quintile being less likely to suffer from anaemia in 2005 (OR = 0.6; [0.3–1.0]), whereas numbers of 2016 showed that anaemia increased noticeably with poverty (OR = 1.8; [1.2–2.7]). Other variables such as nutrition, infections, treatment, hygiene and sanitation were not significantly associated with anaemia when adjusted for other variables.

**TABLE 3:** Multivariable logistic regression results of child anaemia (*N* = 6,154)

		EDHS 2005 (*N* = 1,011)		EDHS 2011 (*N* = 2,589)		EDHS 2016 (*N* = 2,554)	
Variables		Odds ratio	95% CI	Odds ratio	95% CI	Odds ratio	95% CI
Underlying determinants: socio‐demographic and socioeconomic							
Sex	Male (ref.)						
	Female	0.7	0.5; 1.0	1.1	0.8; 1.3	0.9	0.7; 1.1
Age (child)	6–11	1.7^*^	1.0; 2.8	1.7^*^	1.2; 2.2	1.5^*^	1.1; 2.1
	12–23 (ref.)						
Stunting	No (ref.)						
	Yes	1.2	0.7; 1.9	1.1	0.8; 1.5	1.0	0.7; 1.4
Wasting	No (ref.)						
	Yes	1.3	0.7; 2.4	1.0	0.7; 1.4	1.5	1.0; 2.4
Underweight	No (ref.)						
	Yes	1.6	1.0; 2.7	1.2	0.8; 1.6	1.2	0.8; 1.8
Age (w.)	15–19	3.1^*^	1.2; 7.8	1.3	0.8; 2.3	1.7	0.9; 3.4
	20–34	1.4	0.9; 2.0	1.1	0.8; 1.4	1.2	0.9; 1.7
	35–49 (ref.)						
Anaemia (w.)	No (ref.)						
	Yes	2.1^*^	1.4; 3.2	1.3	0.9; 1.8	1.7^*^	1.2; 2.3
Education (w.)	No (ref.)						
	Primary	1.2	0.7; 1.9	0.9	0.7; 1.1	1.2	0.9; 1.6
	Sec./higher	0.5	0.2; 1.2	0.8	0.4; 1.5	0.7	0.4; 1.1
Rural vs. urban	Pastoralist	1.1	0.7; 1.9	1.8^*^	1.4; 2.4	1.5^*^	1.1; 2.1
	Agrarian (ref.)						
	Cities	1.0	0.5; 2.1	1.2	0.8; 2.0	1.4	0.8; 2.3
Wealth	Poorest	2.0	0.8; 2.7	0.8	0.5; 1.1	1.8^*^	1.2; 2.7
	Poor	1.1	0.6; 2.0	1.1	0.8; 1.5	1.1	0.7; 1.6
	Middle (ref.)						
	Wealthy	0.6^*^	0.3; 1.0	0.9	0.6; 1.3	1.0	0.6; 1.5
	Wealthiest	1.0	0.5; 1.8	0.6	0.4; 1.0	0.8	0.5; 1.3
Immediate determinants: nutritional deficiencies							
Diet	Below 4 (ref.)						
	4 and above	2.7	0.6; 12.0	1.0	0.4; 2.6	1.1	0.7; 1.8
Iron‐rich food	No (ref.)						
	Yes	0.9	0.6; 1.2	0.8	0.6; 1.0	0.8	0.6; 1.0
Immediate determinants: infections							
Fever	No (ref.)						
	Yes	0.9	0.5; 1.4	1.0	0.8; 1.4	1.5	1.0; 2.2
Cough	No (ref.)						
	Yes	0.8	0.5; 1.2	0.8	0.6; 1.1	0.8	0.6; 1.2
Diarrhoea	No (ref.)						
	Yes	1.4	0.9; 2.1	0.9	0.7; 1.2	1.0	0.7; 1.4
Underlying determinants: inadequate prevention/treatment and maternal care							
Deworming	No (ref.)						
	Yes	0.9	0.3; 2.3	1.1	0.8; 1.5	1.7^*^	1.0; 2.7
Immunization	No (ref.)						
	Yes	1.2	0.6; 2.1	0.8	0.6; 1.1	1.1	0.8; 1.5
Vitamin A	No (ref.)						
	Yes	1.0	0.7; 1.5	0.9	0.7; 1.2	1.0	0.7; 1.3
Nr. of births	1 or 2 (ref.)						
	More than 2	1.0	0.5; 2.0	1.1	0.7; 1.6	1.5^*^	0.9; 2.6
Underlying determinants: water, hygiene and sanitation							
Drinking water	Improved (ref.)						
	Unimproved	1.0	0.7; 1.4	1.1	0.9; 1.4	1.0	0.7; 1.3
Toilet facility	Improved (ref.)						
	Unimproved	0.9	0.6; 3.9	1.1	0.7; 1.5	0.9	0.6; 1.449

*Note*: Variables are defined as above; w: women.

* *p* < 0.05.

### 3.4 Concentration index

Table 4 provides information on the concentration indices for the different years. The index values were negative for child anaemia, indicating a higher prevalence of anaemia among the less affluent households. The table depicts a significantly negative value for the year 2005, which indicated a higher concentration of children with anaemia among the poor and a moderate level of inequality (−0.150; [−0.2 to −0.1]). In 2011 the concentration index of −0.019 [−0.1–0.0] confirmed child anaemia was less heavily concentrated among worse‐off households compared with the survey before. However, wealth‐based inequality was found not to be significant. The last survey demonstrated a significant tendency of inequality, shown by an index of −0.094 [−0.1 to −0.1]. The indices were all negative, indicating that children with anaemia were concentrated among the less affluent households. Larger values, as measured in 2005 and 2016, represent moderate levels of inequality. The index value of 2011 was close to zero, implying fewer inequalities existed at that time.

**TABLE 4:** Concentration indices of child anaemia in Ethiopia per year

	EDHS 2005		EDHS 2011		EDHS 2016	
	Con. index	95% CI	Con. index	95% CI	Con. index	95% CI
Child anaemia	−0.150^*^	−0.2; −0.1	−0.019	−0.1; 0.0	−0.094^*^	−0.1; −0.1

*Note*: con. index: concentration index.

* *p* < 0.05.

## 4 DISCUSSION

By investigating the prevalence of anaemia over 11 years, analysing its determinants and inequalities, the results of this study provide a comprehensive overview of the serious public health concern of anaemia among Ethiopian children aged 6–23 months. Previous studies investigated specific determinants, isolated geographical patterns and distinct time‐points of anaemia in Ethiopia (Abebe et al., 2018; Desalegn et al., 2014; Ejigu et al., 2018; Kebede, Gerensea, Amare, Tesfay, & Teklay, 2018; Reithinger et al., 2013). This study offers essential insights on top of these findings. Looking at the results of the multiple analyses, some main findings can be identified.

Anaemia prevalence continues to be alarmingly high, especially in infants younger than 12 months, children whose mothers are anaemic and children growing up in pastoralist regions. Additionally, we showed that wealth‐based inequalities in anaemia prevalence remained constant over the 11 years, with the highest prevalence existing in the poorest part of the population and performing a severe health problem even among the wealthiest.

The first main finding of this study was that the overall prevalence of anaemia in children aged 6–23 months remained alarmingly high throughout the 11 years. Prevalence was consistently greater than 55% and was highest in 2016, at 72%. These numbers are similar to corresponding data published in the EDHSs (CSA & ICF, 2012, 2017; CSA & ORC Macro, 2006). According to the WHO guidelines, this study classifies anaemia as a severe public health problem (≥40%), regardless of region or wealth status (WHO, 2008, 2015). This result also confirms the findings of other studies carried out in some parts of Ethiopia (Alemayehu, Meskele, Alemayehu, & Yakob, 2019; Woldie, Kebede, & Tariku, 2015) and other parts of the world (Nguyen, Scott, Avula, Tran, & Menon, 2018; Uddin et al., 1970).

Secondly, concentration analysis showed moderate inequalities in the distribution of anaemia by wealth, with higher numbers among people from lower socioeconomic groups. A study highlighted the association between poor economic status and food insecurity. Food insecurity, characterized by decreased nutrient intake and poor health, is a predictor for malnutrition, including iron deficiency (Pasricha et al., 2010). Higher prevalence of anaemia in less affluent households may also be attributed to a lack of access to health care services and education and fewer possibilities to purchase nutrient‐rich food (Nankinga & Aguta, 2019; Sunil & Sagna, 2015). This study's finding is in line with other studies in certain parts of Ethiopia (Birhane, Shiferaw, Hagos, & Mohindra, 2014; Woldie et al., 2015) and sub‐Saharan Africa (Harttgen, Klasen, & Vollmer, 2013). Although anaemia prevalence was generally higher among poorer households, wealthy children struggled with severe anaemia too. Ethiopia's implementation of the National Nutrition Program has not yet tackled the magnitude of anaemia, which may be caused by a lack of focus on underlying socioeconomic disparities influencing nutrition (Kennedy et al., 2015). To achieve ultimate successes, a multisectoral approach is needed to improve health sustainably, together with policies, education and agriculture (Ambel et al., 2017), primarily directed towards risk‐groups.

Multivariable logistic regression showed that children aged 6–11 months had significantly higher odds of being anaemic. Research states that by 4 months of age, neonatal iron stores usually reduce by half (Booth & Aukett, 1997), and by 6 months, children have depleted the iron stores present at birth (Lanzkowsky, 2016). For this reason, all infants should start receiving additional sources of iron at the age of 6 months to maintain sufficient haemoglobin concentration. Unlike the recommended 6 months of exclusive breastfeeding by the WHO (2009), the median duration of exclusive breastfeeding in Ethiopia was between two and 3 months, and complementary foods were not initiated in a timely fashion for many children either. Mothers either perform exclusive breastfeeding for too long or stop too early, which both can result in malnutrition (Alemayehu, Haidar, & Habte, 2009; CSA & ICF, 2012, 2017; CSA & ORC Macro, 2006; Setegn et al., 2012). The absorption of haemoglobin is particularly enhanced by a meat‐based diet, including poultry and fish and fruits and vegetables high in Vitamin C. However, especially meat‐based foods are less likely to be fed (Booth & Aukett, 1997; CSA & ICF, 2017; Meinzen‐Derr et al., 2006). Instead, parents start feeding their children with cow‐milk and other dairy products, which are dietary predictors of iron deficiency (Booth & Aukett, 1997; Tassew, Tekle, Belachew, & Adhena, 2019). It is assumed that this is due to misinformation and inadequate education on breastfeeding and complementary feeding. So far, not many studies have focused on young children, let alone infants in the Ethiopian context. More preventative actions should be brought to national and community attention, revolving around the educating on dietary feeding practices and supplementary iron (Booth & Aukett, 1997; Kulwa, Verstraeten, Bouckaert, Kolsteren, & Lachat, 2014).

Finally, we showed that children growing up in pastoralist regions had significantly higher odds of having anaemia than those in agrarian or urban areas. One potential reason is the difference in dietary patterns and habits per region. People living in pastoralist regions, like Afar and Somali, have little diversity in their diet (Workicho et al., 2016). Moreover, cattle, goat and camel milk are some of their main consumption items known for inhibiting iron absorption (Abegaz, Hassen, & Minten, 2018; Demeke & Di Marcantonio, 2013). Workicho et al. (2016) further explain that urban households have a more diversified diet. For example, people living in Addis Ababa benefit from the increasing commercial food flows due to urbanization, better access to iron‐rich diets and increased demand for meat (Worku, Dereje, Minten, & Hirvonen, 2017). Also, children from agrarian regions benefit from more diverse diets, including dietary iron (Tassew et al., 2019). Also, the production and consumption of teff in Ethiopia have drastically increased, especially in urban settings. Agrarian and pastoralist regions, on the other hand, usually consume maize, wheat and sorghum, which contain far less iron than teff (Baye, 2014; Demeke & Di Marcantonio, 2013; Gebru, Remans, & Brouwer, 2018). Another adverse impact on food security is climate change. According to FAO et al. (2018), climate change has shown dramatic effects in the past (FAO et al., 2018), with severe droughts occurring all over Ethiopia, particularly in Afar and Somali (Federal Democratic Republic of Ethiopia, 2018; Lewis, 2017). Droughts affect food supply due to impairments of crop yield and agricultural productivity, leading to increased food insecurity and peaking numbers in childhood anaemia (Malako et al., 2018).

### 4.1 Implications

Anaemia is preventable, but more effort is needed to combat the public health problem. Interventions addressing anaemia need to align its multifactorial set of determinants. Understanding the factors that influence iron deficiency is crucial for tackling anaemia on national and regional levels.

As anaemia has disproportionately affected those children who were younger than 1 year, those whose mothers were anaemic and who were living in pastoralist regions, our study asks for a nationwide multisectoral approach to address anaemia. Based on this, preventative means must involve the health care sector, including maternal and child health, education and agriculture. For example, monitoring and screening of maternal anaemia should be a fixed component of antenatal care. Especially during pregnancy, anaemia is a risk factor for poor birth outcomes such as low haemoglobin concentrations (Engidaye et al., 2019). Mothers should also be educated on the benefits and management of breastfeeding and supported in complementary feeding, in line with the recommendations by the WHO (2009). As for the DHS surveys, we recommend expanding the questionnaire. Because anaemia also peaks in early childhood, questions about nutrition should include all children younger than 5 years and not only the lastborn (Lanzkowsky, 2016). In Ethiopia, programmes such as the National Nutrition Program (Kennedy et al., 2015) and the Sustainable Undernutrition Reduction (Moss et al., 2018) aim to tackle nutrient deficiency. For such programmes to be effective, knowing about the target populations and vulnerable regions is crucial. Our study offers this information and can be used to evaluate whether programmes are adequately implemented; they pay attention to critical determinants and whether they are inclusive for all children living in Ethiopia.

### 4.2 Limitations

We recognize that our study has limitations. First, this study relies on repeated cross‐sectional surveys. During 11 years, various exogenous events such as the drought El Niño and consequently food shortages peaking in 2015/16 or a challenging economic climate might have influenced numbers in a specific survey year (Gari et al., 2017). Second, the DHS data are self‐reported, which might have subconsciously biased our study too. Third, the cross‐sectional nature of the data did not allow us to attribute causality to any of these findings. Lastly, contrary to previous expectations, iron‐rich food did not significantly affect anaemia. This might be due to particular religious or societal reasons, where animal products are only consumed during special occasions or not at all during times of fasting (Haileselassie et al., 2020). Because we do not know whether the questionnaire was taken on a representative day, this may have led to misclassification bias.

## 5 CONCLUSIONS

This study demonstrates the magnitude of anaemia as a severe public health concern in Ethiopia and provides insight into the most critical determinants of anaemia over the past 11 years. Given the lack of anaemia reduction, Ethiopia must apply nationwide and context‐specific programmes, focusing on pastoralist regions, maternal and child health and the agricultural sector. As anaemia is a multifaceted condition, it requires multisectoral efforts that we suggest future research should investigate. By identifying these key determinants, this study provides policymakers and public health researchers with recommendations for future research and essential targets for tackling anaemia.

## CONFLICTS OF INTEREST

The authors declare that they have no conflicts of interest.

## CONTRIBUTIONS

HH conceived and designed the study, performed the statistical analysis, interpreted the results and wrote the manuscript. BSE conceived the research idea, contributed to conceptualizing the design of the study and assisted in data preparation. TDD assisted in data analysis and the interpretation of the results. GJD reviewed the manuscript. MS helped to conceptualize the study design and gave critical comments at different stages of manuscript development. All authors have seen and approved the final version of the manuscript.

## Supporting information


**Table S1a.** Clarification of Variables.
**Figure S2a.** Conceptual framework for anaemia.

## Data Availability

The study used three Demographic and Health Surveys, conducted in 2005, 2011 and 2016. The data can be accessed from http://www.dhsprogram.com/ by registering and requesting the datasets.
