# Supplementary material for: Anaemia and its determinants among young children aged 6–23 months in Ethiopia (2005–2016)
Source: Matern Child Nutr. 2020 Sep 23;17(2):e13082. doi: 10.1111/mcn.13082 (PMC7988878; doi:10.1111/mcn.13082)
Supplement: Supplementary file 1 — Table S1a. Clarification of Variables. Figure S2a. Conceptual framework for anaemia. [file MCN-17-e13082-s001.docx]

**Additional files**

**Additional file 1: Table S1a.** Clarification of Variables. **Figure S2a.** Conceptual framework for anemia.

# *Appendix A:* Clarification of variables

| *Variables, unit of measurement* | ***Type*** | ***Categories*** |
| --- | --- | --- |
| *Outcome* |  |  |
| Children anemia status | Dichotomous | None  Any (mild, moderate, severe) |
| *Immediate determinants: nutritional deficiencies* | | |
| Duration breastfeeding | Ordinal | Never, not currently  Never  Still |
| Flesh food | Dichotomous | No  Yes |
| Legumes | Dichotomous | No  Yes |
| Vegetables and fruits | Dichotomous | No  Yes |
| Milk | Dichotomous | No  Yes |
| Dairy | Dichotomous | No  Yes |
| Iron-rich foods | Dichotomous | No  Yes |
| *Immediate determinants: infections and other inflammatory diseases* | | |
| Fever | Ordinal | No  Yes |
| Cough | Ordinal | No  Yes |
| Diarrhea | Ordinal | No  Yes |
| *Underlying determinants: inadequate prevention/ treatment and maternal/ child care* | | |
| ≥4 ANC visits | Dichotomous | No  Yes |
| >90 IFA supplements during pregnancy | Dichotomous | No  Yes |
| Number of births past 5 years | Dichotomous | 1 or 2  More than 2 |
| Full immunization, child | Dichotomous | No  Yes |
| Received malaria treatment, child | Dichotomous | No  Yes |
| Received vitamin A supplements, child | Dichotomous | No  Yes |
| *Underlying determinants: inadequate sanitation or lack of safe water* | | |
| Drinking water | Dichotomous | Unimproved  Improved |
| Toilet facility | Dichotomous | Unimproved  Improved |
| *Underlying determinants: socio-demographic and -economic* | | |
| Sex | Dichotomous | Male Female |
| Age, months | Nominal | 6-11  12-23 |
| Height-for-age | Ordinal | No  Percentage below -3 SD  Percentage below -2 SD |
| Weight-for-height | Ordinal | No  Percentage below -3 SD  Percentage below -2 SD  Percentage above +2 SD |
| Weight-for-age | Ordinal | No  Percentage below -3 SD  Percentage below -2 SD  Percentage above +2 SD |
| Age women, years | Nominal | 15-19  20-34  35-49 |
| Women anemia status | Dichotomous | No  Yes |
| Mother’s education | Ordinal | No education  Primary  Secondary/ Higher |
| Partner’s education | Ordinal | No education  Primary  Secondary/ Higher |
| Marital status | Ordinal | Married/ living together  Widowed/ divorced  Single |
| Region | Polytomous | Tigray  Afar  Amhara  Oromiya  Somali  Benishangul-Gumuz  SNNPR  Gambela  Harari  Addis Ababa  Dire Dawa |
| Rural vs. urban | Ordinal | Pastoralist  Agrarian  Urban Centers |
| Wealth quintile | Ordinal | Poorest  Poor  Middle  Wealthy  Wealthiest |

# *Appendix B:* Conceptual framework for anemia


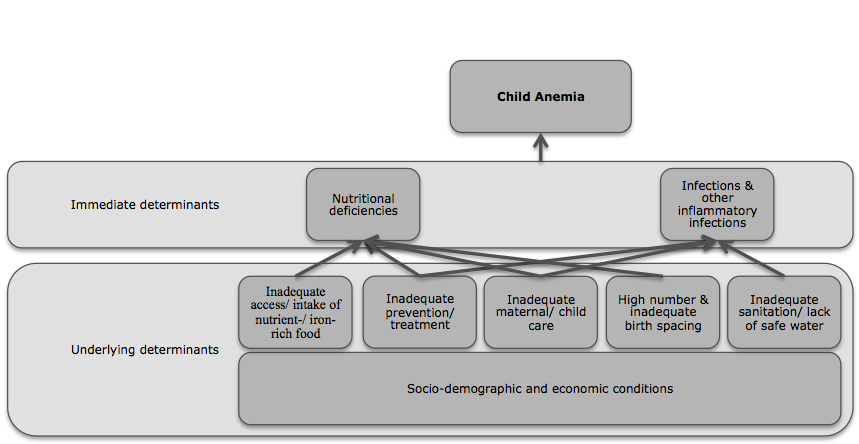


Conceptual framework for the basic, underlying and immediate determinants of anemia in children. This framework was adapted from the USAID Anemia Task Force held on 18 October 2013 (Gray & Mueller, 2012)
